# Gene modification strategies for next-generation CAR T cells against solid cancers

**DOI:** 10.1186/s13045-020-00890-6

**Published:** 2020-05-18

**Authors:** Yonggui Tian, Yilu Li, Yupei Shao, Yi Zhang

**Affiliations:** 1 Biotherapy Center, The First Affiliated Hospital of Zhengzhou University, Zhengzhou, 450052 China; 2 Cancer Center, The First Affiliated Hospital of Zhengzhou University, Zhengzhou, 450052 China; 3 Henan Key Laboratory for Tumor Immunology and Biotherapy, Zhengzhou, 450052 China; 4 School of Medicine, Zhengzhou University, Zhengzhou, 450052 China

**Keywords:** CAR T cells, Immunotherapy, Genetic engineering, Solid tumor

## Abstract

Immunotherapies have become the backbone of cancer treatment. Among them, chimeric antigen receptor (CAR) T cells have demonstrated great success in the treatment of hematological malignancies. However, CAR T therapy against solid tumors is less effective. Antigen targeting; an immunosuppressive tumor microenvironment (TME); and the infiltration, proliferation, and persistence of CAR T cells are the predominant barriers preventing the extension of CAR T therapy to solid tumors. To circumvent these obstacles, the next-generation CAR T cells will require more potent antitumor properties, which can be achieved by gene-editing technology. In this review, we summarize innovative strategies to enhance CAR T cell function by improving target identification, persistence, trafficking, and overcoming the suppressive TME. The construction of multi-target CAR T cells improves antigen recognition and reduces immune escape. Enhancing CAR T cell proliferation and persistence can be achieved by optimizing costimulatory signals and overexpressing cytokines. CAR T cells equipped with chemokines or chemokine receptors help overcome their poor homing to tumor sites. Strategies like knocking out immune checkpoint molecules, incorporating dominant negative receptors, and chimeric switch receptors can favor the depletion or reversal of negative T cell regulators in the TME.

## Background

**Fig. 1:**
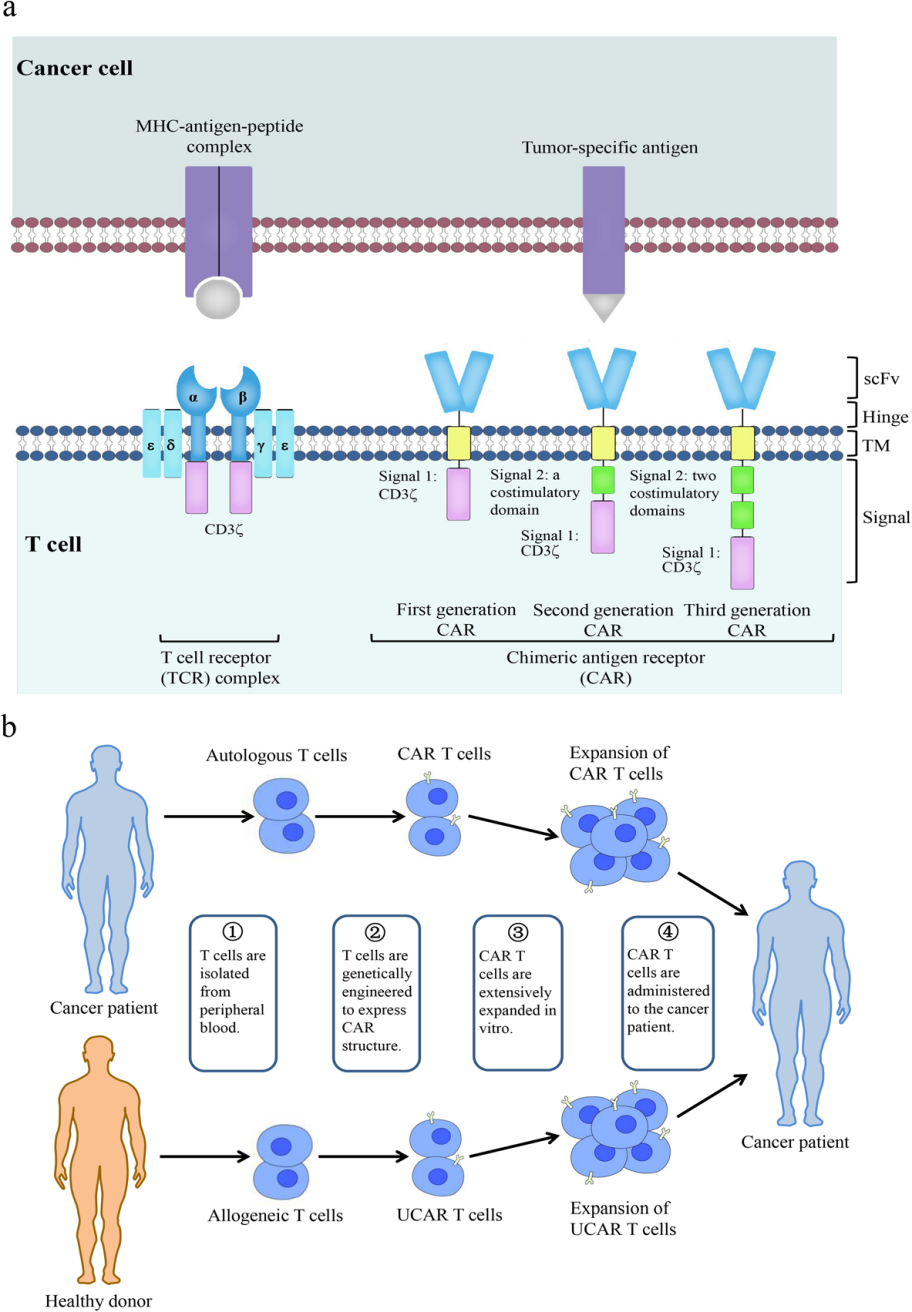
CAR T cell design and treatment. **a** The structure of TCR and CARs. TCR (left) comprises variable α- and β- chains connected to invariant CD3 chains on T cells, which interact with major histocompatibility complex (MHC)-antigen-peptide complexes on cancer cells, to induce the activation of T cells. CARs (right) can recognize tumor-specific antigens in an MHC-unrestricted manner. First-generation CARs contain only one intracellular signaling domain, CD3ζ. One or two costimulatory signaling domains are introduced to construct the second- or third-generation CARs, respectively. **b** CAR T cell therapy. After isolating T cells from the peripheral blood of the patient (autologous T cells, above) or a healthy donor (allogeneic T cells, below), CAR genes are engineered into T cells to generate CAR T cells (above) or UCAR T cells (below), which are then extensively expanded in vitro and administered to the patient

CAR T cells are autologous or allogeneic T cells genetically engineered to express a synthetic chimeric antigen receptor (CAR). These cells are emerging as a promising cancer immunotherapy. A typical CAR has an extracellular single-chain variable fragment (scFv), fused through a transmembrane domain to intracellular costimulatory signaling domains. The recognition of tumor antigens by scFv activates the intracellular costimulatory signaling domain, subsequently stimulating T cell proliferation and eliminating tumor cells through cytolysis and cytokine release. Based on the structure of the intracellular region, CARs are divided into three generations. First-generation CARs contain only one intracellular signaling domain, CD3ζ, to provide signal 1, but this is insufficient to trigger CAR T cell expansion and engender continuous antitumor activity in vivo [1]. In contrast, second- and third-generation CARs utilize first-generation CARs as a backbone and incorporate one or two additional costimulatory signaling domains, such as CD28 or 4-1BB (CD137), respectively [2]. Unlike T cell receptors (TCRs), CARs recognize specific surface targets on tumor cells in a major histocompatibility complex (MHC)-unrestricted manner, effectively preventing immune escape by downregulating MHC expression in tumor cells [3] (Fig. 1a). In addition to peptide antigens, CARs also recognize carbohydrates and glycolipid antigens, thus expanding the range of tumor antigen targets [4].

To date, CAR T cell-based immunotherapy has yielded remarkable clinical success in patients with CD19-positive leukemia or lymphoma [5, 6]. Owning to these results, Kymriah, a CAR T therapy developed by Novartis, has been approved by the US Food and Drug Administration (FDA) and the European Commission for the treatment of relapsed or refractory acute lymphoblastic leukemia in children and young adults. In October 2017, the US FDA permitted Yescarta, another CD19-targeting CAR T cell product, to treat relapsed or difficult-to-treat large B cell lymphoma [7, 8].

However, there remain a series of challenges that need to be urgently tackled in CAR T cell therapy. In the manufacturing process of CAR T cells, most of the currently available CAR T cells are from autologous T cells. Generation of autologous CAR T cells require huge costs and a long production cycle, limiting their large-scale clinical application. Therefore, universal CAR T cells derived from allogeneic healthy donors are created as an “off-the-shelf” product. Graft-versus-host disease can be avoided by abating TCR and human leukocyte antigen class-1 (HLA-I) expression in universal CAR T cells with gene-editing technologies [9] (Fig. 1b). Apart from this, desirable efficacy of CAR T cells has not been achieved in solid tumors, due to multiple limiting factors. Five major challenges of using CAR T cells to treat solid tumors have been summarized [10]. These include the heterogeneity of tumor antigens, toxicity control, inadequate infiltration, poor proliferation and persistence, and the immunosuppressive tumor microenvironment (TME). Due to the heterogeneity of solid tumors, CAR T cells targeting a single antigen may result in tumor recurrence due to excessive growth of antigen-negative cells or cells expressing low antigen levels. The migration of CAR T cells to the tumor site is the primary step in achieving effector function. Nevertheless, insufficient expression of chemokine receptors in CAR T cells and physical barriers inhibit their homing to tumor sites. Costimulatory signals and cytokine production are also associated with CAR T cell function [11]. The absence of costimulatory molecules can lead to CAR T cell inactivation, while the incorporation of costimulatory molecules strengthens CAR T cell function [12]. The presence of cytokines, as signal 3, is a prerequisite for the full activation of CAR T cells, but exhausted CAR T cells have lower levels of cytokine receptor expression and reduced reactivity to inflammatory cytokines [13], causing the abatement of cytotoxic function. In addition, the TME weakens the function of T cells through a variety of factors, such as the upregulated and sustained expression of inhibitory receptors [14], which is the main characteristic of exhausted T cells [15]. These problems highlight the significance of developing new methods to optimize CAR T cell structure and overcome the treatment hurdles raised by solid tumors.

Herein, we outline and analyze several novel strategies for enhancing CAR T cell function, to improve therapeutic responses against solid tumors, including targeting multiple antigens, overexpressing cytokine or chemokine receptors, enhancing costimulatory signals, and eliminating negative T cell regulators. These next-generation CAR T cells have shown promise in preclinical models and they have great prospects for the treatment of solid tumors.

## Improving CAR T cell antigen recognition

Antigen-specific recognition is a prerequisite for effective CAR T cell therapy. To avoid immune escape and improve safety, tumor-associated antigens (TAAs) that are specifically and highly expressed in tumor tissue are usually selected as targets for CAR T cells [16, 17]. However, these TAAs are also expressed in normal tissues and therefore, despite their low levels, off-target cross-reactions still occur with CAR T cell therapy. In addition, in contrast to hematological tumors, solid tumors are characterized by antigen heterogeneity. CAR T cells preferentially kill tumor cells with high expression levels of the targeted antigens, but they may not eliminate cells with lower antigen density [18]. Various studies have shown that CAR T cell treatment results in the down-regulation of targeted antigens, which is one of the reasons for tumor recurrence [19].

To improve CAR T cell antigen recognition, new CAR T cell strategies have been developed to simultaneously target two or more different TAAs or to mix different CAR T cells targeting a single antigen. CAR T cells co-targeting CD19 and HER2 display greater antitumor activity in vivo than CAR T cells with a single target [20]. IL13Rα2- and HER2-specific scFvs together form a bi-specific CAR (TanCAR) [21]. TanCAR can recognize each antigen individually to trigger activation, while its functionality is synergistically enhanced upon encountering both antigens [22]. Similarly, trivalent CAR T cells co-targeting HER2, IL13Rα2, and EphA2 effectively eliminate tumor cells [23]. Numerous studies have demonstrated that bi-specific CAR T cells co-targeting CD19 and either CD20 [24], CD22 [25], or CD133 [26] have the potential to reduce the risk of CD19-negative relapse.

Another multi-target strategy is to connect the scFvs of CD3 and the TAA using a flexible linker, to form bi-specific T cell engagers (BiTEs). BiTE-secreting CAR T cells can target multiple antigens, and also have the ability to activate and recruit bystander T cells [27]. Anti-EGFR BiTEs enhance CAR T cell efficacy in vivo and eliminate glioblastoma [28]. CAR T cells can kill tumor cells in a lower antigen density than BiTEs. And costimulatory molecules such as CD28 and 4-1BB can enhance the effect and killing function of BiTEs-activated T cells. Therefore, compared with BiTE or CAR T cells alone, BiTE-secreting CAR T cells display stronger antitumor ability through direct killing and induced bystander killing. Similarly, the construction of universal CAR T cells using adapters can target multiple TAAs without re-engineering T cells. Recently universal CAR structures, such as biotin-binding immune receptor, binding fluorescein isothiocyanate (anti-FITC CAR) and antibody Fc receptor (CD16 CAR), have been developed [29–31]. And a SUPRA (split, universal, and programmable) CAR system has been developed, which is composed of a leucine zipper CAR (zipCAR) and a scFv binding to the leucine zipper (zipFv). By adjusting zipFv, this approach makes CAR T cells to target multiple antigens, and reduces potential immunogenicity [32]. Compared with traditional CAR T cells, these adapter systems (BiTEs and universal CARs) establish a bridge between CAR T cells and tumor cells, improving the safety and effectiveness of CAR T cells. In addition, the recognition ability of CAR T cells can also be improved by increasing antigen density. The exogenous addition of IL-22 increases the expression of MUC1. CAR-MUC1-IL22 T cells secreting IL-22 enhance recognition and cytotoxic ability against head and neck squamous cell carcinoma [33].

Targeting multiple antigens allows these CAR T cells to maintain their cytotoxicity, expand the range of recognized TAAs, and mitigate antigen escape. The modification of a multi-target CAR to recognize diverse tumor antigens results in more effective elimination of an established tumor than a unispecific CAR. Nevertheless, the combination of multiple targets increases the potential for off-target effects of CAR T cells. Multi-target CAR constructs greatly increase the genetic payload delivered to cells, which leads to reduced transduction efficiency [3]. As transposon-based gene transfer systems for stable genetic modification, Sleeping Beauty and piggyBac transposon systems have attracted much attention. After electroporation, this non-viral approach confers superior CAR expression and is less toxic compared to conventional plasmids [34, 35]. Moreover, optimizing T cell culture conditions and CAR structure have shown to improve transfection efficiency [36]. Therefore, multi-target CAR T cells require further study to improve their structural design and safety.

## Enhancing CAR T cell proliferation and persistence

After homing to the tumor site, CAR T cells must undergo abundant expansion in order to eliminate the tumor in vivo. The major predictors of the clinical efficacy of CAR T cells are the proliferation and persistence of the T cells. Vast clinical trials of CAR T cell therapy have demonstrated poor persistence of T cells transfused in vivo, especially in solid tumors [16]. Therefore, improving the expansion and persistence of CAR T cells is the focus of current research.

### Costimulatory signaling domain optimization

Considerable efforts have been concentrated on the optimization of CAR constructs to enhance costimulatory signaling. The incorporation of one or more costimulatory signaling domains (CSSDs), such as CD28 [37], 4-1BB [38], OX40 [39], CD27 [40], or ICOS [41], can enhance the proliferation, persistence, and effector function of CAR T cells. The selection of costimulatory molecules also impacts the kinetics, tumoricidal profile, and safety of CAR T cells. Therefore, understanding the characteristics of known and emerging costimulatory signal domains is essential for improving the efficacy of CAR T cell therapy.

CD28 and 4-1BB are the most widely tested costimulatory domains in clinical trials [42]. CD28-based CAR T cells are associated with more rapid T cell proliferation and tumor elimination, while CAR T cells expressing the 4-1BB domain possess noticeably slower kinetics, but greater persistence [43]. Compared with CD28, ICOS costimulation induces a greater increase in PI3K activity [44] and drives CAR T cells to differentiate into Th17/Th1 cells [45]. Analogous to 4-1BB-based CARs, CD27-based CARs lead to enhanced persistence compared with CD28-based CARs [40]. The specific combination of costimulatory domains also has an impact on CAR T cell activity. PSMA-CAR T cells bearing both CD28 and 4-1BB display the strong PI3Kinase/Akt activation, relevant to reduced apoptosis and enhanced effector function of CD8^+^ T cells [46]. Although a third generation CD28-OX40-CD3ζ CAR T cell increases the production of IL-2 and IL-10 [39], CAR T cells with a CD28-CD3ζ-OX40 construct show the opposite result [47]. Analogously, ICOS-4-1BB-CD3ζ CAR (ICOSBBz) produces more IL-7 and IL-10, whereas the reverse CAR (BBICOSz) produces more IL-6 and IL-13, suggesting that the membrane-proximal domain is of great importance in determining cytokine secretion [48]. In order to further explore the functional characteristics and clinical efficacy, various clinical trials on the second or third generation CARs above have been carried out, involving multiple antigen targets and tumor types [49] (Table 1).

**Table 1:** Summary of costimulatory molecules in CAR T cell trials for solid tumors

Costimulatory molecules	Functional characteristics	CAR target antigen	Malignancy	Clinical trials	Phase	Status	Enrolled patients	Sponsor
CD28	Increased cytokine production; more potent effector function; more rapid T cell proliferation and tumor elimination [43]	Lewis Y	Advanced solid cancer	NCT03851146	I	Recruiting	30	Peter MacCallum Cancer Centre
		Chlorotoxin	Glioma	NCT04214392	I	Recruiting	36	City of Hope Medical Center
		HER2	Sarcoma	NCT00902044	I	Active, not recruiting	36	Baylor College of Medicine
			Glioblastoma	NCT01109095	I	Completed	16	Baylor College of Medicine
4-1BB	Greater persistence; increased central memory T cell generation [43]	PSCA	Prostate cancer	NCT03873805	I	Recruiting	33	City of Hope Medical Center
		PD-L1	Lung cancer	NCT03330834	I	Recruiting	22	Sun Yat-sen University
		Mesothelin	Pancreatic cancer	NCT03638193	I	Recruiting	10	Shenzhen BinDeBio Ltd.
			MPM	NCT01355965	I	Completed	18	University of Pennsylvania
			Ovarian cancer	NCT02159716	I	Completed	19	University of Pennsylvania
		GPC3	Hepatocellular cancer	NCT02715362	I/II	Recruiting	30	Shanghai GeneChem Co Ltd.
		IL13Ralpha2	Melanoma	NCT04119024	I	Recruiting	24	Jonsson Comprehensive Cancer Center
			Glioblastoma	NCT04003649	I	Recruiting	60	City of Hope Medical Center
		CEA	Liver metastases	NCT02862704	I/II	Recruiting	20	Xijing Hospital
		CD171	Neuroblastoma	NCT02311621	I	Recruiting	40	Seattle Children’s Hospital
		MET	Melanoma	NCT03060356	I	Terminated	77	University of Pennsylvania
			Breast cancer	NCT03060356	I	Terminated	77	University of Pennsylvania
		EGFRvIII	Glioblastoma	NCT03726515	I	Active, not recruiting	7	University of Pennsylvania
CD28 and 4-1BB	Strong PI3Kinase/Akt activation; reduced apoptosis of CD8^+^ T cells; enhanced effector function of CD8^+^ T cells [46]	CD171	Neuroblastoma	NCT02311621	I	Recruiting	40	Seattle Children's Hospital
CD28 and OX40	CD28-OX40-CD3ζ CAR increased the production of IL-2 and IL-10; CD28-CD3ζ-OX40 CAR decreased the production of IL-2 and IL-10 [39, 47]	GD2	Neuroblastoma	NCT01822652	I	Active, not recruiting	11	Baylor College of Medicine
			Sarcoma	NCT01953900	I	Active, not recruiting	26	Baylor College of Medicine

*CAR* chimeric antigen receptor, *HER2* human epidermal growth factor receptor-2, *PSCA* prostate stem cell antigen, *GPC3* glypican-3, *CEA* carcinoembryonic antigen, *MET* mesenchymal to epithelial transition factor, *EGFRvIII* epidermal growth factor receptor variant III, *MPM* malignant pleural mesothelioma, *GD2* disialoganglioside2

In addition to known costimulatory molecules, other more novel costimulatory molecules are under active exploration. There is accumulating evidence demonstrating a critical role of herpesvirus entry mediator (HVEM, TNFRSF14) in the memory development of T cells [50]. CAR T cells bearing an HVEM-derived CSSD display the greater effector function than those with CD28- or 4-1BB-derived CSSD, which may be due to the reduced T cell exhaustion, reprogrammed energy metabolism and balanced differentiation of memory T cell subsets [51]. Toll-like receptor 2 (TLR2) is known to strengthen the effector function and proliferation of CD8^+^ T cells and reduce the activation threshold of costimulatory signaling [52, 53]. By adding TLR2 to the 3′ end of m28z CAR (m28zT2 CAR), the resulting CAR T cells exhibit enhanced cytotoxicity and expansion capacity and lower expression levels of exhaustion markers [54]. However, various degrees of cytokine release syndrome (CRS) occurred in patients receiving 1928zT2 CAR T cells [55], suggesting that more clinical trials are required to monitor their side effects. Natural killer group 2 member D is a strong costimulatory receptor expressed on NK and CD8^+^ T cells [56]. It can transmit an activating signal in T cells, via the adaptor protein, DNAX-activating protein 10 (DAP10), leading to memory formation and enhanced inflammatory cytokine production in CD8^+^ T cells [57, 58]. Thus, DAP10 incorporation into the 3′ end of CAR indeed improves the antitumor activity of CAR T cells against lung cancer, hepatocellular carcinoma, and gastric cancer in mouse models [59, 60]. Both TLR2 and DAP10 incorporation can elevate the expression of T-bet, a transcription factor mediating T cell differentiation, which provides a direction for exploring new costimulatory molecules. Collectively, these findings underscore the importance of optimizing costimulatory molecules in CAR T cells.

Thus, the CSSD is crucial for modulating CAR T cell activity. More significantly, elucidating the mechanistic and biological differences of costimulatory molecules and determining the optimal CSSD combination will be a top priority in future studies.

### Cytokine strategy

Optimal T cell activation, amplification, and persistence require not only antigen engagement (signal 1) and costimulatory signals (signal 2), but also cytokine support (signal 3). However, signal 3 is deficient in the TME, which hampers the full activation of T cells [61]. Therefore, constructing CAR T cells that can provide signal 3 will help promote their activation and proliferation.

#### Transgenic cytokine expression

Initially, the exogenous administration of cytokines to cancer patients was put into clinical testing, but it was found to induce adverse events [62]. To minimize systemic toxicity and induce the accumulation of high cytokine concentrations at the tumor site, T cells redirected for universal cytokine killing (TRUCKs) were developed. TRUCKs are engineered with a nuclear factor of activated T cells (NFAT)-responsive promoter that drives cytokine secretion, only when the CAR encounters a tumor antigen. The cytokines, IL-12, IL-18, IL-7, IL-15, and IL-21 have been extensively studied using this strategy.

IL-12 and IL-18 play a major role in augmenting the effector function of CAR T cells. IL-12 is known to activate NK cells and T lymphocytes, induce Th-1 type responses, and increase IFN-γ secretion [63, 64]. The inducible expression of IL-12 augments the antitumor capability of CAR T cells against lymphoma, hepatocellular carcinoma, ovarian tumors, and B16 melanoma in mouse models [65–68]. IL-18 has also been used to improve the therapeutic potential of CAR T cells. Initially identified as a potent inducer of IFN-γ, IL-18 contributes to T and NK cell activation and Th-1 cell polarization [69]. Meso-targeted CAR T cells delivering IL-18 augment the secretion of IFN-γ and show enhanced proliferative ability to eradicate cancer cells [70]. Furthermore, IL-18-releasing CAR T cells convert to T-bet^high^FoxO1^low^ effectors, with superior activity against solid tumors [71]. However, IL-18 may participate in promoting tumor angiogenesis, metastasis, and immune escape [72] and thus, there is a risk of tumor progression when using IL-18-expressing CAR T cells in clinical trials.

IL-7, IL-15, and IL-21 belong to the common γ chain cytokine family [73]. They promote the generation of the stem cell-like memory T cell (Tscm) phenotype, which is associated with increased expansion and persistence [74–78]. Recently, 7 × 19 CAR T cells, which are engineered to co-express IL-7 and CCL19, prolong persistence compared with conventional CAR T cells, resulting in complete control of tumor progression [79]. Similarly, GD2 CAR T cells expressing IL-15 retain a Tscm phenotype and have lower expression levels of exhaustion markers, like PD-1 and LAG3 [80]. GPC3 CAR T cells co-expressing IL-15 and IL-21 exhibit the most superior proliferation and sustained persistence compared with either cytokine alone or controls [81].

It is worth emphasizing that in this strategy, the combined expression of cytokines may have the potential to maximize the function of CAR T cells, suggesting future directions for CAR T cell modification. In addition, other more promising cytokines, such as IL-27, IL-10, and IL-23, also warrant in-depth investigation. For instance, IL-27 induces a memory precursor cell phenotype in cytotoxic T lymphocytes, by stimulating high levels of IL-10 production [82]. IL-10 promotes the generation of memory T cells and also markedly improves their cytotoxicity [82]. Moreover, IL-23 promotes the proliferation of memory T cells [83]. In a recent study reported by Ma and colleagues, CAR T cells engineered with the IL-23 p40 subunit showed increased granzyme B secretion and decreased PD-1 expression levels and superior antitumor ability [84]. Therefore, further studies are required for a greater understanding of the effect of cytokines on T cells. This information will lay the groundwork for the next-generation CAR T cell modifications. At last, considering that high tumor burden is likely to over-activate TRUCKs, thereby releasing tremendous amounts of cytokines, which may enter circulation and produce toxicity. Therefore, more attention should be paid to monitor the risk of side effects. Meanwhile, larger-scope clinical trials are required to evaluate the safety and efficacy of TRUCKs.

#### Constitutive IL-7 cytokine receptor signaling

Because transgenic cytokine expression carries a latent risk of adverse events, Shum et al. [85] proposed a safer approach to deliver signal 3 to CAR T cells without the participation of exogenous cytokines. They constructed an IL-7 cytokine receptor (C7R) that activated STAT5, the downstream signaling molecule of IL-7, in an antigen-dependent manner. Therefore, C7R had a unique ability to provide signal 3 only to CAR T cells, without affecting bystander lymphocytes. In addition, C7R expression can significantly improve the activation, proliferation, and persistence of CAR T cells, to effectively eliminate triple-negative breast cancer cells [86].

#### CAR with JAK/STAT signaling

**Fig. 2:**
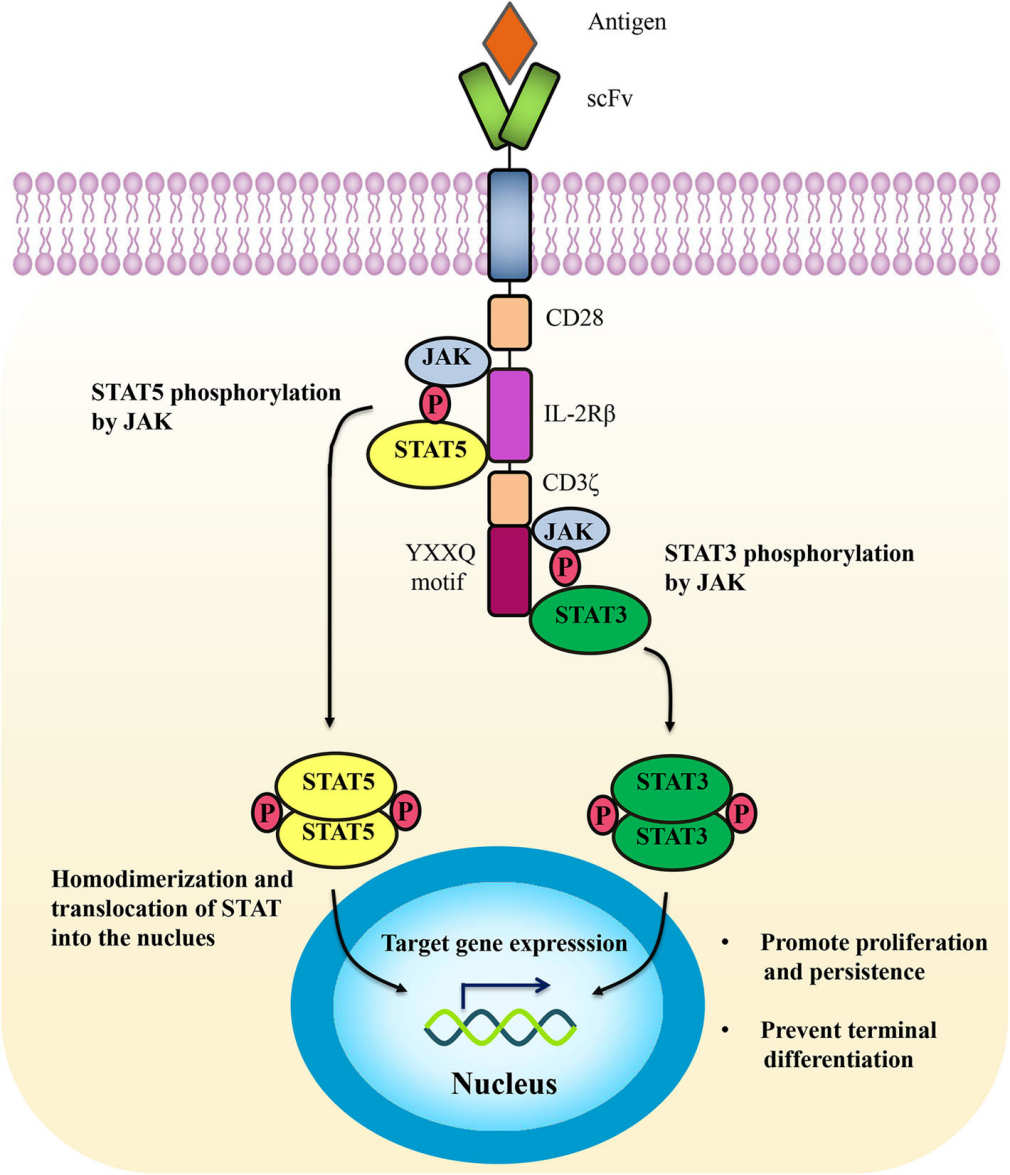
CAR with JAK/STAT signaling. The 28-IL2RB-z(YXXQ) CAR induces the phosphorylation of JAK kinases after antigen engagement, resulting in the phosphorylation of STAT3 and STAT5. Phosphorylated STAT3 (pSTAT3) and STAT5 (pSTAT5) subsequently homodimerize and translocate into the nucleus to regulate the transcription of target genes, which leads to the enhanced proliferation and reduced terminal differentiation of CAR T cells.

Both of the strategies described above highlight the significance of transmitting signal 3 to CAR T cells. The activation of JAK/STAT signaling is mediated by γc family cytokines, such as IL-7, IL-15, and IL-21, which exert a profound influence on the expansion, differentiation, development, and survival of lymphocytes [87]. A novel CAR construct is developed to activate JAK/STAT signaling after antigen engagement. The new CAR, denoted 28-IL2RB-z(YXXQ), incorporates a truncated cytoplasmic domain of IL-2Rβ for STAT5 recruitment and a STAT3-binding YXXQ motif in the co-stimulation domain [88] (Fig. 2). 28-IL2RB-z(YXXQ) CAR T cells demonstrate superior proliferation and reduced terminal differentiation in vitro and in vivo. These findings reveal a pivotal role of the STAT3 pathway in promoting memory T cell differentiation and maintenance, in line with previous studies [89, 90]. Moreover, high STAT3 activity in T cells is correlated with favorable clinical outcomes [91], indicating that STAT3 activation may be an effective strategy to prolong CAR T cell persistence.

## Increasing CAR T cell trafficking to the tumor site

**Fig. 3:**
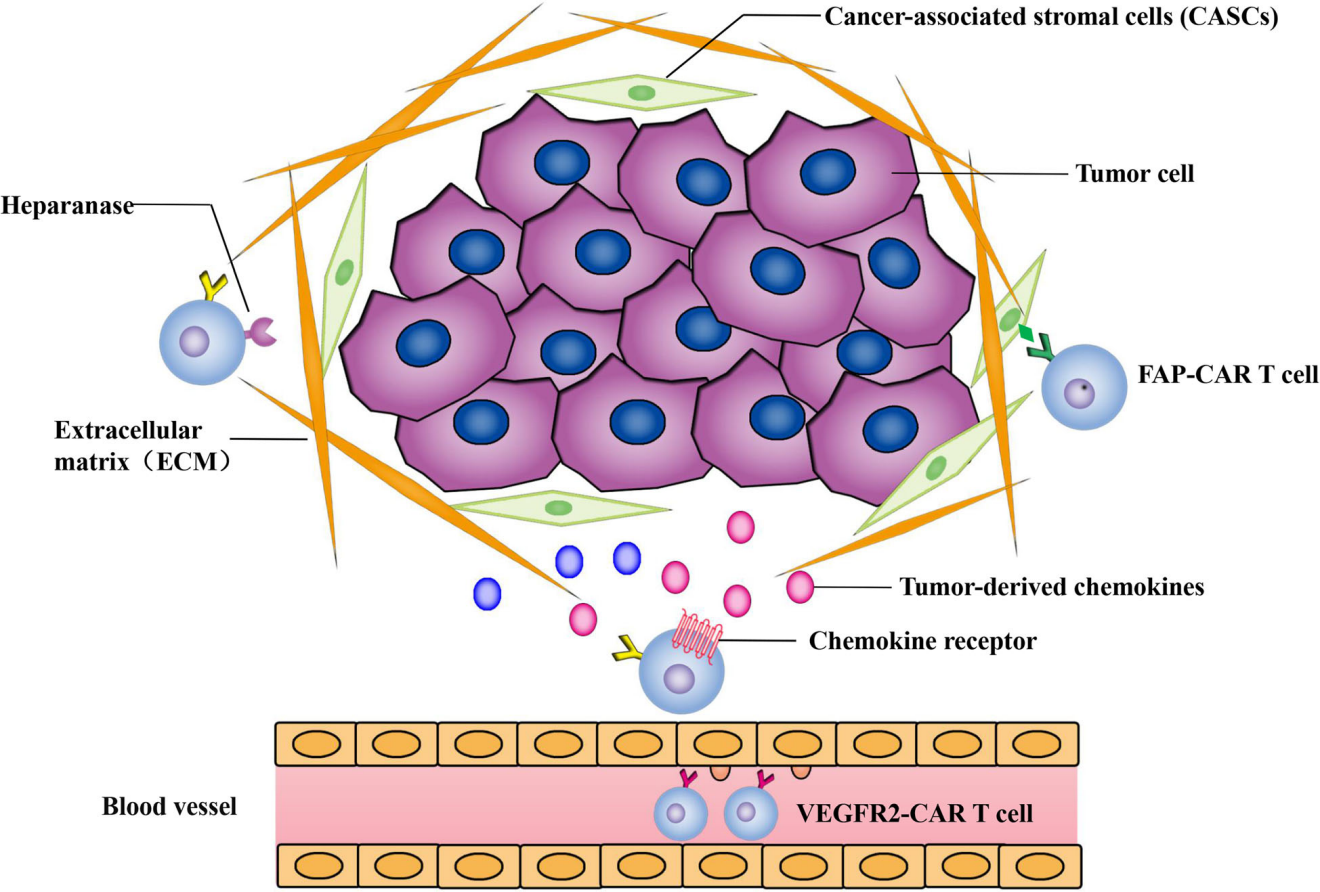
Increasing CAR T cell trafficking to the tumor site. Tumor cells in the tumor microenvironment (TME) can secrete large quantities of chemokines. Identifying the most highly secreted chemokines in the targeted tumor and overexpression of the corresponding chemokine receptors on CAR T cells can improve their trafficking ability to tumor sites. Tumor blood vessels and extracellular matrix (ECM) are the main physical barriers hindering the infiltration of CAR T cells. VEGFR2-CAR T cells can destroy tumor vascular endothelial cells to increase penetration. FAP-CAR T cells can inhibit stromagenesis and angiogenesis by targeting FAP^+^ CASCs. CAR T cells expressing heparanase can degrade heparan sulfate proteoglycan to disrupt the ECM

The unremarkable efficacy of CAR T cells against solid tumors is associated with insufficient intratumoral T cell penetration and accumulation [92]. Improving the homing of CAR T cells to the TME is a prerequisite for them to exert therapeutic activity. To successfully infiltrate and accumulate in tumor sites, CAR T cells undergo a series of processes, involving the adhesion of endothelial cells and interactions between chemokines and chemokine receptors [93] (Fig. 3). Although the local infusion of CAR T cells results in the significant regression of glioblastoma, this approach may not be effective for all tumor types, especially metastatic disease [94].

Chemokines and chemokine receptors play a pivotal role in mediating the directed migration of CAR T cells. Thus, equipping T cells with a chemokine receptor to better match and respond to tumor-derived chemokines may facilitate T cell migration ability. Integrin αvβ6-CAR T cells modified to express CXCR2 migrate more efficiently towards tumor-produced IL-8 [95]. Similarly, CAR T cells overexpressing CXCR1 or CXCR2 can significantly reduce tumor burden, without obvious toxicity, in various solid tumor xenografts [96, 97]. Further, forced expression of CCR4 by CD30 CAR T cells in a Hodgkin lymphoma model, enhances their homing to Reed-Stemberg cells secreting CCL17 and CCL22, the ligand of CCR4 [98]. Meso-targeted CAR T cells with CCR2b transduction result in increased migration and tumoricidal effect [99]. Furthermore, CXCR3 has been shown to be essential for the intravascular adhesion and penetration of adoptively transferred CD8^+^ T cells [100]. The expression of CXCL9, CXCL10, and CXCL11 (ligands of CXCR3), increased by chemotherapy drugs, enhances T cell trafficking to the TME [101], suggesting that CXCR3 is also a candidate for chemokine receptor modification of CAR T cells. Identifying an appropriate chemokine-chemokine receptor axis is of cardinal importance, but it requires numerous studies to confirm a specific chemokine with high expression levels in the targeted tumor and to overexpress the corresponding receptors on CAR T cells. The incorporation of a chemokine receptor does not affect the cytotoxicity of CAR T cells but exert superior antitumor activity. Of note, chemokines recruit not only effector T cells but also suppressor cells like regulatory T cells [102]. Moreover, factors like tumor types, stroma, and ambient cytokines may affect the tumor’s chemokine signature. Therefore, these aspects need to be taken into account when choosing the suitable chemokine receptor for CAR T cell constructs.

Engineering CAR T cells to deliver chemokines locally is another feasible strategy to recruit more immune cells into the tumor lesion. For instance, forced expression of CCL19 substantially enhances the chemotactic capability of CD20 CAR T cells, by inducing T cell and DC cell migration [79]. Co-expression of CCL5 and CXCL9 is key to CD8^+^ T cell recruitment, creating a loop to magnify lymphocyte engraftment [103]. However, it should be noted that the production of endogenous chemokines in CAR T cells may block chemokine receptors on their surface, resulting in attenuated migratory ability.

Other obstacles, such as the tumor blood vessels and extracellular matrix (ECM), also hinder the infiltration of CAR T cells [104]. Vascular endothelial growth factor receptor 2 (VEGFR2) plays a vital role in VEGF-mediated tumor angiogenesis and growth. CAR T cells targeting VEGFR2 display increased penetration and antitumor responses, by destroying tumor vascular endothelial cells [105]. Enhancing the trafficking of CAR T cells can also be achieved by targeting fibroblast activation protein (FAP), which is a surface marker of cancer-associated stromal cells (CASCs) [106]. FAP-CAR T cells can inhibit stromagenesis and angiogenesis by targeting FAP^+^ CASCs [107]. However, FAP-CAR T cells cause serious cachexia and bone toxicities by affecting FAP^+^ bone marrow stromal cells [108]. And the affinity and specificity of the FAP scFv deserve more attention to decrease the toxicity profile of this modification. Analogous to CASCs, heparan sulfate proteoglycan is an indispensable component of the ECM that can be degraded by heparanase. GD2 CAR T cells expressing heparanase enhance infiltration capacity and antitumor activity in a neuroblastoma xenograft model [109]. It is worth noting that intravenous injection of ECM-degrading enzymes in patients leads to increased thromboembolic events, so precaution like the addition of low molecular weight heparin need to be adopted.

## Overcoming CAR T cell dysfunction

There is increasing evidence that CAR T cells administered to patients gradually lose their effector function and fail to eliminate tumors, due to the TME and internal T cell factors [16, 110, 111]. T cell effector function is regulated by various factors, such as immune checkpoint molecules, transcription factors, metabolic molecules, and apoptotic genes [112]. Therefore, regulating the factors that impact T cell function may enhance the efficacy of CAR T cells (Table 2).

**Table 2:** Knockout of negative regulators in CAR T cells improves antitumor activity

Negative regulators	Name	Malignancy	Genome editing tool	Function	Reference(s)
Immune checkpoint molecules	PD-1	ALL, CML, TNBC, HCC, Glioma	CRISPR-Cas9, TALEN, AAV–Cpf1	Improved cytokine production, infiltration and persistence of CAR T cells; enhanced tumor clearance	[113–119]
	CTLA-4	NMIBC, CML	CRISPR-Cas9	Improved CAR T cell function	[119, 120]
	LAG3	Burkitt lymphoma, CML	CRISPR-Cas9	Improved CAR T cell function	[121]
Transcription factors	NR4A	Melanoma, Thymoma, COAD	—	Promoted tumor regression	[122]
	TOX	Melanoma, COAD	shRNAs	Augmented antitumor responses	[123]
Metabolic molecules	DGKs	GBM	CRISPR-Cas9	Increased TCR signaling in CAR T cells; enhanced CAR T cell effector function	[124]
Apoptotic genes	Fas	ALL, CML	CRISPR-Cas9	Increased tolerance of CAR T cells to apoptosis	[119]

*AAV* adeno-associated virus, *ALL* acute lymphoblastic leukemia, *CAR* chimeric antigen receptor, *COAD* colon adenocarcinoma, *CML* chronic myeloid leukemia, *shRNA* short hairpin RNA, *NMIBC* non-muscle invasive bladder cancer, *TALEN* transcription activator-like effector nuclease, *TNBC* triple-negative breast cancer, *HCC* hepatocellular carcinoma, *GBM* glioblastoma

### Knocking out negative T cell regulators

#### Immune checkpoint molecules

Unlike hematological malignancies, a major problem with solid tumors is the presence of an immunosuppressive TME [10, 125]. In the TME, tumor cells activate immune checkpoint receptors (PD-1, CTLA-4, LAG3, TIGIT, VISTA) on T cells, through the expression of their ligands [3, 126] and deliver immunosuppressive signals, which drive T cells into a state of exhaustion, tolerance, or dysfunction [127, 128]. Therefore, blocking the co-suppression signal in CAR T cells may reverse the exhausted phenotype of CAR T cells and improve their antitumor function [129]. Powerful techniques, such as CRISPR/Cas9, TALEN, and AAV-Cpf1 platforms, have recently emerged as efficient strategies to eradicate the immune checkpoint molecules, PD-1, CTLA-4, and LAG3 [113, 120, 121, 130].

Numerous studies have demonstrated that CRISPR/Cas9-mediated PD-1 disruption enhances the antitumor activity of CAR T cells in orthotopic mouse models of chronic myeloid leukemia, triple-negative breast cancer, hepatocellular carcinoma, and glioma [114–117]. The deletion of PD-1 improves cytokine production and the infiltration and persistence of CAR T cells. Moreover, PD-1-deficient CAR T cells enhance tumor clearance and relapse prevention. Dai et al. [113] and Gautron et al. [118] used AAV–Cpf1 and TALEN, respectively, to integrate CAR into TCRα constant chain (TRAC) and knock out PD-1. The simultaneous disruption of TRAC and PD-1 augments CAR T cell stability, to prevent adverse events, and enhances the efficacy of CAR T therapy. Similarly, the disruption of LAG3 or CTLA-4 can improve CAR T cell function [120, 121]. Using a one-shot CRISPR system simultaneously ablates four genes (*HLA-I*, *TCR*, *PD1*, and *CTLA-4*) to generate universal CAR T cells with both PD1 and CTLA-4 disruption [119]. The simultaneous blockade of the PD-1 and CTLA-4 inhibitory pathways may enhance CAR T cell function. However, the efficiency of gene disruption and transfection in CAR T cells is low, because the gRNAs competed for Cas9 and the packaging size of the lentivirus is limited. And the deletion of PD-1 gene leads to accumulation of T cell exhaustion during chronic viral infection. Therefore, the complete deficiency of immune checkpoints in CAR T cells may drive the terminal differentiation of T cells.

#### Transcription factors

Transcription factors play critical roles in the T cell-mediated immune response [131]. The NR4A family of nuclear receptor transcription factors includes NR4A1, NR4A2, and NR4A3 [132]. NR4A receptors are induced by NFAT, and are constitutively active receptors that do not depend on ligand engagement [132, 133]. It also has been shown that CD8^+^ T cells expressing PD-1 and TIM3 in the TME display chromatin accessibility and gene expression, which are correlated with the activation of NR4A receptors [134]. Triple-NR4A-knockout CAR T cells decrease the expression levels of CCR7, PD-1, and TIM3, which induces tumor regression and prolonged the survival of tumor-bearing mice [122]. The thymocyte selection-associated high-mobility group box (TOX) transcription factors are important in regulating the differentiation program of thymocytes [135]. A series of studies have reported that TOX proteins are critical transcriptional regulators of T cell exhaustion [136–138]. TOX and TOX2 express at high levels in CAR T cells with an exhausted phenotype [123]. Thus, CAR T cells with both TOX and TOX2 knocked out exhibite more effective antitumor responses than TOX^-/-^ or TOX2^-/-^ CAR T cells. A variety of transcription factors strictly regulate T cell dysfunction. Inhibiting the expression of exhaustion-related transcription factors in CAR T cells has the potential to prevent tumor-induced exhaustion, which provides large opportunities for tumor immunotherapy.

#### Other molecules

T cells are also regulated by metabolic molecules and apoptotic genes. Diacylglycerol kinases (DGKs) are a class of enzymes that catabolize diacylglycerols (DAGs) [139]. DAG is an essential downstream molecule of the TCR, and it plays an important role in T cell signaling [140]. There are three known DGK isoforms, DGKα, DGKδ, and DGKζ. T cells mainly express DGKα and DGKζ [124, 140]. Using CRISPR/Cas9 to knock out both DGKα and DGKζ increase TCR signaling in CAR T cells (DGK dKO CAR T) [124]. DGK dKO CAR T cells significantly induce glioblastoma regression through enhanced effector function in a xenograft mouse model. Metabolism can affect T cell fate and function. Tumor cells compete with T cells for metabolic substances, which will greatly impact the antitumor ability of T cells. And improving CAR T cell function by controlling cell metabolism is an important modification strategy. The Fas receptor (APO-1/CD95) is a type I transmembrane glycoprotein belonging to the NGF/TNF family. It is widely distributed in various tissues and can induce apoptosis by binding to its ligand, FasL. Fas promotes the invasiveness and motility of multiple cancer cell types, and knockdown of Fas or FasL reduces tumor cell growth and induces cell death [141]. Fas/FasL also induces T cell apoptosis, which affects the therapeutic outcome. Therefore, triple gene disruption in CAR T cells, knocking out *TCR*, *HLA-I*, and *Fas* have been performed [119]. The universal CAR T cells with Fas ablation are resistant to apoptosis and enhance tumor control capability.

### Dominant negative receptor

Forced expression of a dominant negative receptor (DNR) on the surface of CAR T cells is another feasible approach to overcome tumor-derived inhibition. To block immunosuppression mediated by the PD-1/PD-L1 axis, a PD-1 DNR is developed, merely composed of the extracellular PD-L1-binding domain, without the transmembrane and intracellular signaling domains. M28z CAR T cells engineered with the PD-1 DNR augment expansion, cytokine production, and cytotoxicity, and exhibit greater tumor control [142–144]. Transforming growth factor-β (TGF-β), a potent repressor of T lymphocyte function, is expressed abundantly in a variety of solid tumors, particularly prostate cancer. To shield CAR T cells from TGF-β-induced immune inhibition, a dominant negative TGF-β type II receptor (dnTGF-βRII) is generated. CAR T cells expressing dnTGF-βRII exhibit enhance antitumor efficacy in the treatment of prostate cancer [145, 146]. Moreover, CAR T cells engineered with a Fas DNR display superior antitumor activity against solid tumors, due to the disruption of apoptotic signaling by FasL in the TME [147]. The inhibitory signaling pathways that restrict T cell function in the TME have not been fully elucidated so far. Further endeavor needs to be performed to illuminate the potential immunosuppressive mechanism. And blocking multiple inhibitory signaling pathways through DNR strategies may achieve greater clinical benefit.

### Chimeric switch receptor

Chimeric switch receptors (CSRs) reverse the suppressive impact of inhibitory molecules on CAR T cells, by converting negative signals into positive signals. IL-4 is present at elevated levels in multiple solid tumors [148]. It plays a crucial role in promoting tumor progression, by increasing the resistance of tumor cells to apoptosis and by downregulating Th1-polarized T cell responses [149]. Thus, an IL-4 CSR has been constructed composed of the IL-4 receptor ectodomain linked to the IL-7 receptor endodomain (4/7 ICR) or the IL-21 receptor endodomain (4/21 ICR). CAR T cells with 4/7 ICR or 4/21 ICR augment cytolytic function and proliferative potential upon exposure to IL-4 and its cognate antigen [150–153]. CAR T cells expressing 4/7 ICR maintain their Th1 phenotype, whereas 4/21 ICR-CAR T cells display a Th17-polarized profile, with attenuated exhaustion [153]. Downstream signaling triggered by IL-7 and IL-21 receptors may account for this difference. Immune checkpoint molecule CSRs have also been studied successively. These consist of the extracellular portion of inhibitory costimulatory molecules (PD-1, CTLA-4, etc.) and intracellular costimulation domains [154]. CAR T cells with PD-1/CD28 increase resistance to inhibitory signals from PD-L1 and improve antitumor activity [155–157]. Moreover, CTLA-4/CD28 exerts an effect on T cells, similar to the effect of PD-1/CD28 [158, 159]. Because the types and concentrations of inhibitory molecules in the TME may vary in different solid tumors, the chosen CSR should specifically tailor to the characteristic of the TME. Additionally, CSR is primarily exerting enhancing effect through its intracellular domain, thus, other more promising endodomains need to be widely tested in the CSR design.

## Conclusions

**Fig. 4:**
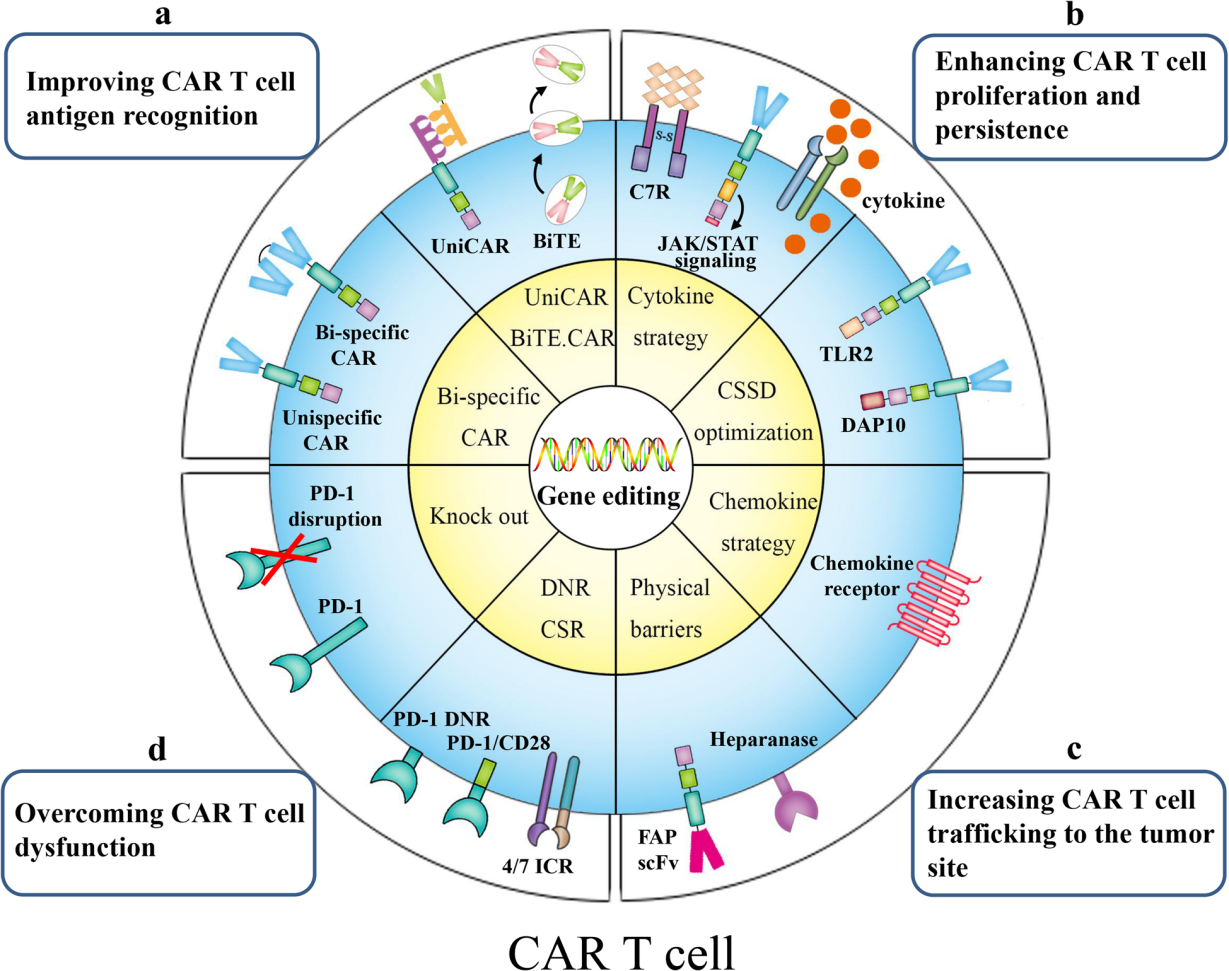
Gene modification strategies for next-generation CAR T cells. **a** Improving CAR T cell antigen recognition: Bi-specific CAR (TanCAR) T cells that target two different TAAs display superior antigen-recognition ability. Universal CAR T cells(UniCAR) and CART.BiTE (BiTE.CAR) utilize adapters connecting CAR T cells and tumor cells, to target multiple antigens. **b** Enhancing CAR T cell proliferation and persistence: C7R activates the downstream IL-7 signaling pathway without the participation of exogenous cytokine. The 28-IL2RB-z(YXXQ) CAR activates the JAK/STAT signaling pathway after antigen engagement, by introducing IL-2Rβ and the tyrosine-X-X-glutamine (YXXQ) motif. Inducible cytokine-secreting CAR T cells release cytokines upon CAR recognition of the tumor-specific antigen. Incorporating costimulatory molecules, such as TLR2 and DAP10, into the 3′ end of CAR augments costimulatory signaling in CAR T cells. **c** Increasing CAR T cell trafficking to the tumor site: The expression of chemokine receptor like CXCR2 in CAR T cells enhances their migratory ability towards tumor-derived chemokines. CAR T cells targeting FAP or expressing heparanase can disrupt physical barriers to increase CAR T cell infiltration. **d** Overcoming CAR T cell dysfunction: Chimeric switch receptors (CSRs), such as 4/7 ICR and PD-1/CD28, transform negative signaling into positive signaling by modifying the receptor endodomain. Dominant-negative receptors (DNRs) without transmembrane or intracellular signaling domains block negative signaling mediated by PD-1. Knocking out the *PD-1* gene using gene-editing technology, such as CRISPR/Cas9, can block the suppression signal in CAR T cells

T cells engineered with CARs are ushering in a new era in cancer immunotherapy. Despite their spectacular success against hematological malignancies, CAR T cells are not effective for the treatment of solid tumors. CAR T cells play a role as combatants in the battlefront of tumor immunity, and their function directly determines therapeutic efficacy. In this review, we discussed external and internal factors that primarily impact CAR T cell function. Moreover, we summarized novel strategies developed to modify CAR T cells to improve their function. Targeting multiple TAAs improves the recognition ability of CAR T cells and reduces immune escape. CSSD optimization has been employed to strengthen costimulatory signaling (signal 2). Additional signal 3 can be provided by transgenic expression of cytokine, cytokine receptor or downstream factors for the optimal activation, proliferation, and persistence of CAR T cells. The overexpression of chemokine receptors on CAR T cells overcomes the obstacles of poor trafficking to tumor sites. Manipulating negative regulators in CAR T cells, such as the depletion of immune checkpoint molecules, helps to regain the effector response. Collectively, these approaches all contribute to exploiting the therapeutic potential of CAR T cells (Fig. 4).

However, adverse reactions, such as CRS and neurological toxicities, some of which can be fatal, have been reported in almost all clinical trials of CAR T cell therapy. The modification of CAR T cells can increase the effects of these adverse events. Therefore, the toxicity of CAR T cells must be taken into consideration. Furthermore, the implementation of additional clinical trials is greatly needed to determine whether the optimal CAR T cell modification can serve as a standard treatment option for patients with solid tumors. Thus, tremendous research efforts are required in the near future. Despite the current obstacles, optimized next-generation CAR T cells have the potential to eventually evolve into an efficient immunotherapy for solid tumors.

## Data Availability

Not applicable.

## Abbreviations

CAR: Chimeric antigen receptor
TME: Tumor microenvironment
TCR: T cell receptor
HLA-I: Human leukocyte antigen class-1
scFv: Single-chain variable fragment
MHC: Major histocompatibility complex
FDA: Food and Drug Administration
TAA: Tumor-associated antigen
TanCAR: Tandem CAR
BiTE: Bi-specific T cell engager
CSSD: Costimulatory signaling domain
HVEM: Herpesvirus entry mediator
TLR2: Toll-like receptor 2
CRS: Cytokine release syndrome
DAP10: DNAX-activating protein 10
TRUCKs: T cell redirected for universal cytokine killing
NFAT: Nuclear factor of activated T cells
Tscm: Stem cell-like memory T cells
C7R: Constitutively signaling IL-7 cytokine receptor
ECM: Extracellular matrix
VEGFR2: Vascular endothelial growth factor receptor 2
FAP: Fibroblast activation protein
CASCs: Cancer-associated stromal cells
CRISPR/Cas9: sgRNA-guided clustered regularly interspaced short palindrome repeats-associated nuclease Cas9
TALEN: Transcription activator-like effector nuclease
AAV-Cpf1: Adeno-associated virus-Cpf1
TRAC: TCRα constant chain
DGK: Diacylglycerol kinase
DAG: Diacylglycerol
DNR: Dominant-negative receptor
TGF-β: Transforming growth factor-β
dnTGF-βRII: Dominant-negative TGF-β typeIIreceptor
CSR: Chimeric switch receptor
HER2: Human epidermal growth factor receptor-2
PSCA: Prostate stem cell antigen
GPC3: Glypican-3
CEA: Carcinoembryonic antigen
MET: Mesenchymal to epithelial transition factor
EGFRvIII: Epidermal growth factor receptor variant III
MPM: Malignant pleural mesothelioma
GD2: Disialoganglioside2
ALL: Acute lymphoblastic leukemia
CML: Chronic myeloid leukemia
TNBC: Triple-negative breast cancer
HCC: Hepatocellular carcinoma
NMIBC: Non-muscle invasive bladder cancer
COAD: Colon adenocarcinoma
GBM: Glioblastoma

## References

[1] Li J, Li W, Huang K, Zhang Y, Kupfer G, Zhao Q (2018). Chimeric antigen receptor T cell (CAR-T) immunotherapy for solid tumors: lessons learned and strategies for moving forward. J Hematol Oncol.

[2] Hay KA, Turtle CJ (2017). Chimeric Antigen Receptor (CAR) T cells: lessons learned from targeting of CD19 in B-cell malignancies. Drugs..

[3] Labanieh L, Majzner RG, Mackall CL (2018). Programming CAR-T cells to kill cancer. Nat Biomed Eng.

[4] Zhang H, Ye Z-L, Yuan Z-G, Luo Z-Q, Jin H-J, Qian Q-J (2016). New strategies for the treatment of solid tumors with CAR-T cells. Int J Biol Sci.

[5] Hirayama AV, Gauthier J, Hay KA, Voutsinas JM, Wu Q, Pender BS, Hawkins RM, Vakil A, Steinmetz RN, Riddell SR (2019). High rate of durable complete remission in follicular lymphoma after CD19 CAR-T cell immunotherapy. Blood..

[6] Cao J, Wang G, Cheng H, Wei C, Qi K, Sang W, Zhenyu L, Shi M, Li H, Qiao J (2018). Potent anti-leukemia activities of humanized CD19-targeted Chimeric antigen receptor T (CAR-T) cells in patients with relapsed/refractory acute lymphoblastic leukemia. Am J Hematol.

[7] Grupp SA, Kalos M, Barrett D, Aplenc R, Porter DL, Rheingold SR, Teachey DT, Chew A, Hauck B, Wright JF (2013). Chimeric antigen receptor-modified T cells for acute lymphoid leukemia. N Engl J Med.

[8] Subklewe M, von Bergwelt-Baildon M, Humpe A (2019). Chimeric antigen receptor t cells: a race to revolutionize cancer therapy. Transfus Med Hemother.

[9] Zhao J, Lin Q, Song Y, Liu D (2018). Universal CARs, universal T cells, and universal CAR T cells. J Hematol Oncol.

[10] Lim WA, June CH (2017). The principles of engineering immune cells to treat cancer. Cell..

[11] Maimela NR, Liu S, Zhang Y. Fates of CD8+ T cells in tumor microenvironment. Comput Struct Biotechnol J. 2019;17. 10.1016/j.csbj.2018.11.004 PMC6297055 30581539

[12] Weinkove R, George P, Dasyam N, McLellan AD (2019). Selecting costimulatory domains for chimeric antigen receptors: functional and clinical considerations. Clin Transl Immunol.

[13] Ingram JT, Yi JS, Zajac AJ (2011). Exhausted CD8 T cells downregulate the IL-18 receptor and become unresponsive to inflammatory cytokines and bacterial co-infections. PLoS Pathog.

[14] Thommen DS, Schumacher TN (2018). T cell dysfunction in cancer. Cancer Cell.

[15] Oelkrug C, Ramage JM (2014). Enhancement of T cell recruitment and infiltration into tumours. Clin Exp Immunol.

[16] Guedan S, Ruella M, June CH (2019). Emerging cellular therapies for cancer. Annu Rev Immunol.

[17] Tokarew N, Ogonek J, Endres S, von Bergwelt-Baildon M, Kobold S (2019). Teaching an old dog new tricks: next-generation CAR T cells. Br J Cancer.

[18] Wing A, Fajardo CA, Posey AD, Shaw C, Da T, Young RM, Alemany R, June CH, Guedan S (2018). Improving CART-cell therapy of solid tumors with oncolytic virus-driven production of a bispecific T-cell Engager. Cancer Immunol Res.

[19] O'Rourke DM, Nasrallah MP, Desai A, Melenhorst JJ, Mansfield K, Morrissette JJD, Martinez-Lage M, Brem S, Maloney E, Shen A (2017). A single dose of peripherally infused EGFRvIII-directed CAR T cells mediates antigen loss and induces adaptive resistance in patients with recurrent glioblastoma. Sci Transl Med.

[20] Grada Z, Hegde M, Byrd T, Shaffer DR, Ghazi A, Brawley VS, Corder A, Schönfeld K, Koch J, Dotti G (2013). TanCAR: a novel bispecific chimeric antigen receptor for cancer immunotherapy. Mol Ther Nucleic Acids.

[21] Hegde M, Mukherjee M, Grada Z, Pignata A, Landi D, Navai SA, Wakefield A, Fousek K, Bielamowicz K, Chow KKH (2016). Tandem CAR T cells targeting HER2 and IL13Rα2 mitigate tumor antigen escape. J Clin Invest.

[22] Schmidts A, Maus MV (2018). Making CAR T cells a solid option for solid tumors. Front Immunol.

[23] Bielamowicz K, Fousek K, Byrd TT, Samaha H, Mukherjee M, Aware N, Wu M-F, Orange JS, Sumazin P, Man T-K (2018). Trivalent CAR T cells overcome interpatient antigenic variability in glioblastoma. Neuro-oncology..

[24] Martyniszyn A, Krahl A-C, André MC, Hombach AA, Abken H (2017). CD20-CD19 Bispecific CAR T Cells for the Treatment of B-Cell Malignancies. Hum Gene Ther.

[25] Jia H, Wang Z, Wang Y, Liu Y, Dai H, Tong C, Guo Y, Guo B, Ti D, Han X (2019). Haploidentical CD19/CD22 bispecific CAR-T cells induced MRD-negative remission in a patient with relapsed and refractory adult B-ALL after haploidentical hematopoietic stem cell transplantation. J Hematol Oncol.

[26] Li D, Hu Y, Jin Z, Zhai Y, Tan Y, Sun Y, Zhu S, Zhao C, Chen B, Zhu J (2018). TanCAR T cells targeting CD19 and CD133 efficiently eliminate MLL leukemic cells. Leukemia..

[27] Choi BD, Kuan C-T, Cai M, Archer GE, Mitchell DA, Gedeon PC, Sanchez-Perez L, Pastan I, Bigner DD, Sampson JH (2013). Systemic administration of a bispecific antibody targeting EGFRvIII successfully treats intracerebral glioma. Proc Natl Acad Sci U S A.

[28] Choi BD, Yu X, Castano AP, Bouffard AA, Schmidts A, Larson RC, Bailey SR, Boroughs AC, Frigault MJ, Leick MB (2019). CAR-T cells secreting BiTEs circumvent antigen escape without detectable toxicity. Nat Biotechnol.

[29] Lohmueller JJ, Ham JD, Kvorjak M, Finn OJ (2017). mSA2 affinity-enhanced biotin-binding CAR T cells for universal tumor targeting. Oncoimmunology..

[30] Lee YG, Marks I, Srinivasarao M, Kanduluru AK, Mahalingam SM, Liu X, Chu H, Low PS (2019). Use of a single CAR T cell and several bispecific adapters facilitates eradication of multiple antigenically different solid tumors. Cancer Res.

[31] Caratelli S, Sconocchia T, Arriga R, Coppola A, Lanzilli G, Lauro D, Venditti A, Del Principe MI, Buccisano F, Maurillo L (2017). FCgamma chimeric receptor-engineered t cells: methodology, advantages, limitations, and clinical relevance. Front Immunol.

[32] Cho JH, Collins JJ, Wong WW (2018). Universal chimeric antigen receptors for multiplexed and logical control of T cell responses. Cell..

[33] Mei Z, Zhang K, Lam AK-Y, Huang J, Qiu F, Qiao B, Zhang Y (2020). MUC1 as a target for CAR-T therapy in head and neck squamous cell carinoma. Cancer Med.

[34] Monjezi R, Miskey C, Gogishvili T, Schleef M, Schmeer M, Einsele H, Ivics Z, Hudecek M (2017). Enhanced CAR T-cell engineering using non-viral Sleeping Beauty transposition from minicircle vectors. Leukemia..

[35] Zheng Y, Li Z-R, Yue R, Fu Y-L, Liu Z-Y, Feng H-Y, Li J-G, Han S-Y (2019). PiggyBac transposon system with polymeric gene carrier transfected into human T cells. Am J Transl Res.

[36] Morita D, Nishio N, Saito S, Tanaka M, Kawashima N, Okuno Y, Suzuki S, Matsuda K, Maeda Y, Wilson MH (2018). Enhanced expression of anti-CD19 chimeric antigen receptor in transposon-engineered T cells. Mol Ther Methods Clin Dev.

[37] Condomines M, Arnason J, Benjamin R, Gunset G, Plotkin J, Sadelain M (2015). Tumor-targeted human T cells expressing CD28-based chimeric antigen receptors circumvent CTLA-4 inhibition. PLoS One.

[38] Long AH, Haso WM, Shern JF, Wanhainen KM, Murgai M, Ingaramo M, Smith JP, Walker AJ, Kohler ME, Venkateshwara VR (2015). 4-1BB costimulation ameliorates T cell exhaustion induced by tonic signaling of chimeric antigen receptors. Nat Med.

[39] Pulè MA, Straathof KC, Dotti G, Heslop HE, Rooney CM, Brenner MK (2005). A chimeric T cell antigen receptor that augments cytokine release and supports clonal expansion of primary human T cells. Mol Ther.

[40] Song D-G, Ye Q, Poussin M, Harms GM, Figini M, Powell DJ (2012). CD27 costimulation augments the survival and antitumor activity of redirected human T cells in vivo. Blood..

[41] Frigault MJ, Lee J, Basil MC, Carpenito C, Motohashi S, Scholler J, Kawalekar OU, Guedan S, McGettigan SE, Posey AD (2015). Identification of chimeric antigen receptors that mediate constitutive or inducible proliferation of T cells. Cancer Immunol Res.

[42] Sadelain M, Brentjens R, Rivière I (2013). The basic principles of chimeric antigen receptor design. Cancer Discov.

[43] Zhao Z, Condomines M, van der Stegen SJC, Perna F, Kloss CC, Gunset G, Plotkin J, Sadelain M (2015). Structural Design of Engineered Costimulation Determines Tumor Rejection Kinetics and Persistence of CAR T Cells. Cancer Cell.

[44] Fos C, Salles A, Lang V, Carrette F, Audebert S, Pastor S, Ghiotto M, Olive D, Bismuth G, Nunès JA (2008). ICOS ligation recruits the p50alpha PI3K regulatory subunit to the immunological synapse. J Immunol.

[45] Guedan S, Chen X, Madar A, Carpenito C, McGettigan SE, Frigault MJ, Lee J, Posey AD, Scholler J, Scholler N (2014). ICOS-based chimeric antigen receptors program bipolar TH17/TH1 cells. Blood..

[46] Zhong X-S, Matsushita M, Plotkin J, Riviere I, Sadelain M (2010). Chimeric antigen receptors combining 4-1BB and CD28 signaling domains augment PI3kinase/AKT/Bcl-XL activation and CD8+ T cell-mediated tumor eradication. Mol Ther.

[47] Hombach AA, Heiders J, Foppe M, Chmielewski M, Abken H (2012). OX40 costimulation by a chimeric antigen receptor abrogates CD28 and IL-2 induced IL-10 secretion by redirected CD4(+) T cells. Oncoimmunology..

[48] Guedan S, Posey AD, Shaw C, Wing A, Da T, Patel PR, Mc Gettigan SE, Casado-Medrano V, Kawalekar OU, Uribe-Herranz M, et al. Enhancing CAR T cell persistence through ICOS and 4-1BB costimulation. JCI Insight. 2018;3(1). 10.1172/jci.insight.96976 PMC5821198 29321369

[49] MacKay M, Afshinnekoo E, Rub J, Hassan C, Khunte M, Baskaran N, Owens B, Liu L, Roboz GJ, Guzman ML (2020). The therapeutic landscape for cells engineered with chimeric antigen receptors. Nat Biotechnol.

[50] Desai P, Abboud G, Stanfield J, Thomas PG, Song J, Ware CF, Croft M, Salek-Ardakani S (2017). J Immunol.

[51] Nunoya J-I, Masuda M, Ye C, Su L (2019). Chimeric antigen receptor T cell bearing herpes virus entry mediator co-stimulatory signal domain exhibits high functional potency. Mol Ther Oncolytics.

[52] Zhang E, Ma Z, Li Q, Yan H, Liu J, Wu W, Guo J, Zhang X, Kirschning CJ, Xu H (2019). TLR2 Stimulation increases cellular metabolism in CD8 T cells and thereby enhances cd8 t cell activation, function, and antiviral activity. J Immunol.

[53] Chapman NM, Bilal MY, Cruz-Orcutt N, Knudson C, Madinaveitia S, Light J, Houtman JCD (2013). Distinct signaling pathways regulate TLR2 co-stimulatory function in human T cells. Cell Signal.

[54] Lai Y, Weng J, Wei X, Qin L, Lai P, Zhao R, Jiang Z, Li B, Lin S, Wang S (2018). Toll-like receptor 2 costimulation potentiates the antitumor efficacy of CAR T Cells. Leukemia..

[55] Weng J, Lai P, Qin L, Lai Y, Jiang Z, Luo C, Huang X, Wu S, Shao D, Deng C (2018). A novel generation 1928zT2 CAR T cells induce remission in extramedullary relapse of acute lymphoblastic leukemia. J Hematol Oncol.

[56] Prajapati K, Perez C, Rojas LBP, Burke B, Guevara-Patino JA (2018). Functions of NKG2D in CD8 T cells: an opportunity for immunotherapy. Cell Mol Immunol.

[57] Perez C, Prajapati K, Burke B, Plaza-Rojas L, Zeleznik-Le NJ, Guevara-Patino JA (2019). NKG2D signaling certifies effector CD8 T cells for memory formation. J Immunother Cancer.

[58] Whitman E, Barber A (2015). NKG2D receptor activation of NF-κB enhances inflammatory cytokine production in murine effector CD8(+) T cells. Mol Immunol.

[59] Zhao R, Cheng L, Jiang Z, Wei X, Li B, Wu Q, Wang S, Lin S, Long Y, Zhang X (2019). DNAX-activating protein 10 co-stimulation enhances the anti-tumor efficacy of chimeric antigen receptor T cells. Oncoimmunology..

[60] Lv J, Zhao R, Wu D, Zheng D, Wu Z, Shi J, Wei X, Wu Q, Long Y, Lin S (2019). Mesothelin is a target of chimeric antigen receptor T cells for treating gastric cancer. J Hematol Oncol.

[61] Chi X, Yang P, Zhang E, Gu J, Xu H, Li M, Gao X, Li X, Zhang Y, Xu H (2019). Significantly increased anti-tumor activity of carcinoembryonic antigen-specific chimeric antigen receptor T cells in combination with recombinant human IL-12. Cancer Med.

[62] Conlon KC, Lugli E, Welles HC, Rosenberg SA, Fojo AT, Morris JC, Fleisher TA, Dubois SP, Perera LP, Stewart DM (2015). Redistribution, hyperproliferation, activation of natural killer cells and CD8 T cells, and cytokine production during first-in-human clinical trial of recombinant human interleukin-15 in patients with cancer. J Clin Oncol.

[63] Waldmann TA (2018). Cytokines in Cancer Immunotherapy. Cold Spring Harb Perspect Biol.

[64] Zundler S, Neurath MF (2015). Interleukin-12: Functional activities and implications for disease. Cytokine Growth Factor Rev.

[65] Kueberuwa G, Kalaitsidou M, Cheadle E, Hawkins RE, Gilham DE (2018). CD19 CAR T Cells Expressing IL-12 Eradicate Lymphoma in Fully Lymphoreplete Mice through Induction of Host Immunity. Mol Ther Oncolytics.

[66] Chinnasamy D, Yu Z, Kerkar SP, Zhang L, Morgan RA, Restifo NP, Rosenberg SA (2012). Local delivery of interleukin-12 using T cells targeting VEGF receptor-2 eradicates multiple vascularized tumors in mice. Clin Cancer Res.

[67] Liu Y, Di S, Shi B, Zhang H, Wang Y, Wu X, Luo H, Wang H, Li Z, Jiang H (2019). Armored Inducible Expression of IL-12 Enhances antitumor activity of glypican-3-targeted chimeric antigen receptor-engineered T cells in hepatocellular carcinoma. J Immunol.

[68] Koneru M, Purdon TJ, Spriggs D, Koneru S, Brentjens RJ (2015). IL-12 secreting tumor-targeted chimeric antigen receptor T cells eradicate ovarian tumors. Oncoimmunology..

[69] Kaplanski G (2018). Interleukin-18: Biological properties and role in disease pathogenesis. Immunol Rev.

[70] Hu B, Ren J, Luo Y, Keith B, Young RM, Scholler J, Zhao Y, June CH (2017). Augmentation of antitumor immunity by human and mouse CAR T cells secreting IL-18. Cell Rep.

[71] Chmielewski M, Abken H (2017). CAR T Cells Releasing IL-18 Convert to T-Bet FoxO1 Effectors that Exhibit Augmented Activity against Advanced Solid Tumors. Cell Rep.

[72] Park S, Cheon S, Cho D (2007). The dual effects of interleukin-18 in tumor progression. Cell Mol Immunol.

[73] Leonard WJ, Lin J-X, O'Shea JJ (2019). The γ Family of cytokines: basic biology to therapeutic ramifications. Immunity..

[74] Cieri N, Camisa B, Cocchiarella F, Forcato M, Oliveira G, Provasi E, Bondanza A, Bordignon C, Peccatori J, Ciceri F (2013). IL-7 and IL-15 instruct the generation of human memory stem T cells from naive precursors. Blood..

[75] Xu Y, Zhang M, Ramos CA, Durett A, Liu E, Dakhova O, Liu H, Creighton CJ, Gee AP, Heslop HE (2014). Closely related T-memory stem cells correlate with in vivo expansion of CAR.CD19-T cells and are preserved by IL-7 and IL-15. Blood..

[76] Zhou J, Jin L, Wang F, Zhang Y, Liu B, Zhao T (2019). Chimeric antigen receptor T (CAR-T) cells expanded with IL-7/IL-15 mediate superior antitumor effects. Protein Cell.

[77] Gargett T, Brown MP (2015). Different cytokine and stimulation conditions influence the expansion and immune phenotype of third-generation chimeric antigen receptor T cells specific for tumor antigen GD2. Cytotherapy..

[78] Chen Y, Yu F, Jiang Y, Chen J, Wu K, Chen X, Lin Y, Zhang H, Li L, Zhang Y (2018). Adoptive transfer of interleukin-21-stimulated Human CD8+ T memory stem cells efficiently inhibits tumor growth. J Immunother.

[79] Adachi K, Kano Y, Nagai T, Okuyama N, Sakoda Y, Tamada K (2018). IL-7 and CCL19 expression in CAR-T cells improves immune cell infiltration and CAR-T cell survival in the tumor. Nat Biotechnol.

[80] Chen Y, Sun C, Landoni E, Metelitsa L, Dotti G, Savoldo B (2019). Eradication of neuroblastoma by T cells redirected with an optimized GD2-specific chimeric antigen receptor and interleukin-15. Clin Cancer Res.

[81] Batra SA, Rathi P, Guo L, Courtney AN, Fleurence J, Balzeau J, Shaik RS, Nguyen TP, Wu M-F, Bulsara S, et al. Glypican-3-Specific CAR T Cells Coexpressing IL15 and IL21 have superior expansion and antitumor activity against hepatocellular carcinoma. Cancer Immunol Res. 2020. 10.1158/2326-6066.CIR-19-0293 PMC10765595 31953246

[82] Liu Z, Liu J-Q, Talebian F, Wu L-C, Li S, Bai X-F (2013). IL-27 enhances the survival of tumor antigen-specific CD8+ T cells and programs them into IL-10-producing, memory precursor-like effector cells. Eur J Immunol.

[83] Li Y, Wang H, Lu H, Hua S (2016). Regulation of Memory T Cells by Interleukin-23. Int Arch Allergy Immunol.

[84] Ma X, Shou P, Smith C, Chen Y, Du H, Sun C, Porterfield Kren N, Michaud D, Ahn S, Vincent B, et al. Interleukin-23 engineering improves CAR T cell function in solid tumors. Nat Biotechnol. 2020. 10.1038/s41587-019-0398-2 PMC7466194 32015548

[85] Shum T, Omer B, Tashiro H, Kruse RL, Wagner DL, Parikh K, Yi Z, Sauer T, Liu D, Parihar R (2017). Constitutive Signaling from an Engineered IL7 Receptor Promotes Durable Tumor Elimination by Tumor-Redirected T Cells. Cancer Discov.

[86] Zhao Z, Li Y, Liu W, Li X (2020). Engineered IL-7 Receptor Enhances the Therapeutic Effect of AXL-CAR-T Cells on Triple-Negative Breast Cancer. Biomed Res Int.

[87] Lin J-X, Leonard WJ (2018). The Common Cytokine Receptor γ Chain Family of Cytokines. Cold Spring Harb Perspect Biol.

[88] Kagoya Y, Tanaka S, Guo T, Anczurowski M, Wang C-H, Saso K, Butler MO, Minden MD, Hirano N (2018). A novel chimeric antigen receptor containing a JAK-STAT signaling domain mediates superior antitumor effects. Nat Med.

[89] Siegel AM, Heimall J, Freeman AF, Hsu AP, Brittain E, Brenchley JM, Douek DC, Fahle GH, Cohen JI, Holland SM (2011). A critical role for STAT3 transcription factor signaling in the development and maintenance of human T cell memory. Immunity..

[90] Cui W, Liu Y, Weinstein JS, Craft J, Kaech SM (2011). An interleukin-21-interleukin-10-STAT3 pathway is critical for functional maturation of memory CD8+ T cells. Immunity..

[91] Explaining Resistance to CAR T Cells. Cancer Discov. 2018;8(7):784–5. 10.1158/2159-8290.CD-NB2018-065 29769184

[92] Jindal V, Arora E, Gupta S (2018). Challenges and prospects of chimeric antigen receptor T cell therapy in solid tumors. Med Oncol.

[93] Do HTT, Lee CH, Cho J (2020). Chemokines and their receptors: multifaceted roles in cancer progression and potential value as cancer prognostic markers. Cancers.

[94] Brown CE, Alizadeh D, Starr R, Weng L, Wagner JR, Naranjo A, Ostberg JR, Blanchard MS, Kilpatrick J, Simpson J (2016). Regression of glioblastoma after chimeric antigen receptor T-cell therapy. N Engl J Med.

[95] Whilding LM, Halim L, Draper B, Parente-Pereira AC, Zabinski T, Davies DM, Maher J (2019). CAR T-Cells Targeting the Integrin αvβ6 and Co-Expressing the Chemokine Receptor CXCR2 Demonstrate Enhanced Homing and Efficacy against Several Solid Malignancies. Cancers.

[96] Liu G, Rui W, Zheng H, Huang D, Yu F, Zhang Y, Dong J, Zhao X, Lin X. CXCR2-modified CAR-T cells have enhanced trafficking ability that improves treatment of hepatocellular carcinoma. Eur J Immunol. 2020. 10.1002/eji.201948457 31981231

[97] Jin L, Tao H, Karachi A, Long Y, Hou AY, Na M, Dyson KA, Grippin AJ, Deleyrolle LP, Zhang W (2019). CXCR1- or CXCR2-modified CAR T cells co-opt IL-8 for maximal antitumor efficacy in solid tumors. Nat Commun.

[98] Di Stasi A, De Angelis B, Rooney CM, Zhang L, Mahendravada A, Foster AE, Heslop HE, Brenner MK, Dotti G, Savoldo B (2009). T lymphocytes coexpressing CCR4 and a chimeric antigen receptor targeting CD30 have improved homing and antitumor activity in a Hodgkin tumor model. Blood..

[99] Moon EK, Carpenito C, Sun J, Wang L-CS, Kapoor V, Predina J, Powell DJ, Riley JL, June CH, Albelda SM (2011). Expression of a functional CCR2 receptor enhances tumor localization and tumor eradication by retargeted human T cells expressing a mesothelin-specific chimeric antibody receptor. Clin Cancer Res.

[100] Mikucki ME, Fisher DT, Matsuzaki J, Skitzki JJ, Gaulin NB, Muhitch JB, Ku AW, Frelinger JG, Odunsi K, Gajewski TF (2015). Non-redundant requirement for CXCR3 signalling during tumoricidal T-cell trafficking across tumour vascular checkpoints. Nat Commun.

[101] Gao Q, Wang S, Chen X, Cheng S, Zhang Z, Li F, Huang L, Yang Y, Zhou B, Yue D (2019). Cancer-cell-secreted CXCL11 promoted CD8 T cells infiltration through docetaxel-induced-release of HMGB1 in NSCLC. J Immunother Cancer.

[102] Li J, Byrne KT, Yan F, Yamazoe T, Chen Z, Baslan T, Richman LP, Lin JH, Sun YH, Rech AJ (2018). Tumor Cell-Intrinsic Factors Underlie Heterogeneity of Immune Cell Infiltration and Response to Immunotherapy. Immunity..

[103] Dangaj D, Bruand M, Grimm AJ, Ronet C, Barras D, Duttagupta PA, Lanitis E, Duraiswamy J, Tanyi JL, Benencia F (2019). Cooperation between Constitutive and Inducible Chemokines Enables T Cell Engraftment and Immune Attack in Solid Tumors. Cancer Cell.

[104] Tahmasebi S, Elahi R, Esmaeilzadeh A (2019). Solid tumors challenges and new insights of CAR T cell engineering. Stem Cell Rev Rep.

[105] Chinnasamy D, Yu Z, Theoret MR, Zhao Y, Shrimali RK, Morgan RA, Feldman SA, Restifo NP, Rosenberg SA (2010). Gene therapy using genetically modified lymphocytes targeting VEGFR-2 inhibits the growth of vascularized syngenic tumors in mice. J Clin Invest.

[106] Wang L-CS, Lo A, Scholler J, Sun J, Majumdar RS, Kapoor V, Antzis M, Cotner CE, Johnson LA, Durham AC (2014). Targeting fibroblast activation protein in tumor stroma with chimeric antigen receptor T cells can inhibit tumor growth and augment host immunity without severe toxicity. Cancer Immunol Res.

[107] Lo A, Wang L-CS, Scholler J, Monslow J, Avery D, Newick K, O'Brien S, Evans RA, Bajor DJ, Clendenin C (2015). Tumor-promoting desmoplasia is disrupted by depleting FAP-expressing stromal cells. Cancer Res.

[108] Tran E, Chinnasamy D, Yu Z, Morgan RA, Lee C-CR, Restifo NP, Rosenberg SA (2013). Immune targeting of fibroblast activation protein triggers recognition of multipotent bone marrow stromal cells and cachexia. J Exp Med.

[109] Caruana I, Savoldo B, Hoyos V, Weber G, Liu H, Kim ES, Ittmann MM, Marchetti D, Dotti G (2015). Heparanase promotes tumor infiltration and antitumor activity of CAR-redirected T lymphocytes. Nat Med.

[110] Schietinger A, Philip M, Krisnawan VE, Chiu EY, Delrow JJ, Basom RS, Lauer P, Brockstedt DG, Knoblaugh SE, Hämmerling GJ (2016). Tumor-specific T cell dysfunction is a dynamic antigen-driven differentiation program initiated early during tumorigenesis. Immunity..

[111] Yang L, Li A, Lei Q, Zhang Y (2019). Tumor-intrinsic signaling pathways: key roles in the regulation of the immunosuppressive tumor microenvironment. J Hematol Oncol.

[112] Chang JT, Wherry EJ, Goldrath AW (2014). Molecular regulation of effector and memory T cell differentiation. Nat Immunol.

[113] Dai X, Park JJ, Du Y, Kim HR, Wang G, Errami Y, Chen S (2019). One-step generation of modular CAR-T cells with AAV-Cpf1. Nat Methods.

[114] Hu W, Zi Z, Jin Y, Li G, Shao K, Cai Q, Ma X, Wei F (2019). CRISPR/Cas9-mediated PD-1 disruption enhances human mesothelin-targeted CAR T cell effector functions. Cancer Immunol Immunother.

[115] Rupp LJ, Schumann K, Roybal KT, Gate RE, Ye CJ, Lim WA, Marson A (2017). CRISPR/Cas9-mediated PD-1 disruption enhances anti-tumor efficacy of human chimeric antigen receptor T cells. Sci Rep.

[116] Guo X, Jiang H, Shi B, Zhou M, Zhang H, Shi Z, Du G, Luo H, Wu X, Wang Y (2018). Disruption of PD-1 Enhanced the Anti-tumor Activity of Chimeric Antigen Receptor T Cells Against Hepatocellular Carcinoma. Front Pharmacol.

[117] Hu B, Zou Y, Zhang L, Tang J, Niedermann G, Firat E, Huang X, Zhu X (2019). Nucleofection with Plasmid DNA for CRISPR/Cas9-Mediated Inactivation of Programmed Cell Death Protein 1 in CD133-Specific CAR T Cells. Hum Gene Ther.

[118] Gautron A-S, Juillerat A, Guyot V, Filhol J-M, Dessez E, Duclert A, Duchateau P, Poirot L (2017). Fine and Predictable Tuning of TALEN Gene Editing Targeting for Improved T Cell Adoptive Immunotherapy. Mol Ther Nucleic Acids.

[119] Ren J, Zhang X, Liu X, Fang C, Jiang S, June CH, Zhao Y (2017). A versatile system for rapid multiplex genome-edited CAR T cell generation. Oncotarget..

[120] Zhang W, Shi L, Zhao Z, Du P, Ye X, Li D, Cai Z, Han J, Cai J (2019). Disruption of CTLA-4 expression on peripheral blood CD8 + T cell enhances anti-tumor efficacy in bladder cancer. Cancer Chemother Pharmacol.

[121] Zhang Y, Zhang X, Cheng C, Mu W, Liu X, Li N, Wei X, Liu X, Xia C, Wang H (2017). CRISPR-Cas9 mediated LAG-3 disruption in CAR-T cells. Front Med.

[122] Chen J, López-Moyado IF, Seo H, Lio C-WJ, Hempleman LJ, Sekiya T, Yoshimura A, Scott-Browne JP, Rao A (2019). NR4A transcription factors limit CAR T cell function in solid tumours. Nature..

[123] Seo H, Chen J, González-Avalos E, Samaniego-Castruita D, Das A, Wang YH, López-Moyado IF, Georges RO, Zhang W, Onodera A (2019). TOX and TOX2 transcription factors cooperate with NR4A transcription factors to impose CD8 T cell exhaustion. Proc Natl Acad Sci U S A.

[124] Jung I-Y, Kim Y-Y, Yu H-S, Lee M, Kim S, Lee J (2018). CRISPR/Cas9-Mediated Knockout of DGK Improves Antitumor Activities of Human T Cells. Cancer Res.

[125] Mirzaei HR, Pourghadamyari H, Rahmati M, Mohammadi A, Nahand JS, Rezaei A, Mirzaei H, Hadjati J. Gene-knocked out chimeric antigen receptor (CAR) T cells: Tuning up for the next generation cancer immunotherapy. Cancer Lett. 2018;423. 10.1016/j.canlet.2018.03.010 29544719

[126] Eisenberg V, Hoogi S, Shamul A, Barliya T, Cohen CJ (2019). T-cells “à la CAR-T(e)” - Genetically engineering T-cell response against cancer. Adv Drug Deliv Rev.

[127] Speiser DE, Ho P-C, Verdeil G (2016). Regulatory circuits of T cell function in cancer. Nat Rev Immunol.

[128] Davoodzadeh Gholami M, Kardar GA, Saeedi Y, Heydari S, Garssen J, Falak R. Exhaustion of T lymphocytes in the tumor microenvironment: Significance and effective mechanisms. Cell Immunol. 2017;322. 10.1016/j.cellimm.2017.10.002 29079339

[129] Ribas A, Wolchok JD (2018). Cancer immunotherapy using checkpoint blockade. Science..

[130] Menger L, Sledzinska A, Bergerhoff K, Vargas FA, Smith J, Poirot L, Pule M, Hererro J, Peggs KS, Quezada SA (2016). TALEN-Mediated Inactivation of PD-1 in Tumor-Reactive Lymphocytes Promotes Intratumoral T-cell Persistence and Rejection of Established Tumors. Cancer Res.

[131] Thaventhiran JED, Fearon DT, Gattinoni L (2013). Transcriptional regulation of effector and memory CD8+ T cell fates. Curr Opin Immunol.

[132] Sekiya T, Kashiwagi I, Yoshida R, Fukaya T, Morita R, Kimura A, Ichinose H, Metzger D, Chambon P, Yoshimura A (2013). Nr4a receptors are essential for thymic regulatory T cell development and immune homeostasis. Nat Immunol.

[133] Martinez GJ, Pereira RM, Äijö T, Kim EY, Marangoni F, Pipkin ME, Togher S, Heissmeyer V, Zhang YC, Crotty S (2015). The transcription factor NFAT promotes exhaustion of activated CD8^+^ T cells. Immunity..

[134] Mognol GP, Spreafico R, Wong V, Scott-Browne JP, Togher S, Hoffmann A, Hogan PG, Rao A, Trifari S (2017). Exhaustion-associated regulatory regions in CD8 tumor-infiltrating T cells. Proc Natl Acad Sci U S A.

[135] Yao C, Sun H-W, Lacey NE, Ji Y, Moseman EA, Shih H-Y, Heuston EF, Kirby M, Anderson S, Cheng J (2019). Single-cell RNA-seq reveals TOX as a key regulator of CD8 T cell persistence in chronic infection. Nat Immunol.

[136] Khan O, Giles JR, McDonald S, Manne S, Ngiow SF, Patel KP, Werner MT, Huang AC, Alexander KA, Wu JE (2019). TOX transcriptionally and epigenetically programs CD8 T cell exhaustion. Nature..

[137] Alfei F, Kanev K, Hofmann M, Wu M, Ghoneim HE, Roelli P, Utzschneider DT, von Hoesslin M, Cullen JG, Fan Y (2019). TOX reinforces the phenotype and longevity of exhausted T cells in chronic viral infection. Nature..

[138] Scott AC, Dündar F, Zumbo P, Chandran SS, Klebanoff CA, Shakiba M, Trivedi P, Menocal L, Appleby H, Camara S (2019). TOX is a critical regulator of tumour-specific T cell differentiation. Nature..

[139] Eichmann TO, Lass A (2015). DAG tales: the multiple faces of diacylglycerol--stereochemistry, metabolism, and signaling. Cell Mol Life Sci.

[140] Riese MJ, Moon EK, Johnson BD, Albelda SM (2016). Diacylglycerol Kinases (DGKs): Novel Targets for Improving T Cell Activity in Cancer. Front Cell Dev Biol.

[141] Peter ME, Hadji A, Murmann AE, Brockway S, Putzbach W, Pattanayak A, Ceppi P (2015). The role of CD95 and CD95 ligand in cancer. Cell Death Differ.

[142] Chen N, Morello A, Tano Z, Adusumilli PS (2017). CAR T-cell intrinsic PD-1 checkpoint blockade: A two-in-one approach for solid tumor immunotherapy. Oncoimmunology..

[143] Huang X, Yang Y (2016). Driving an improved CAR for cancer immunotherapy. J Clin Invest.

[144] Cherkassky L, Morello A, Villena-Vargas J, Feng Y, Dimitrov DS, Jones DR, Sadelain M, Adusumilli PS (2016). Human CAR T cells with cell-intrinsic PD-1 checkpoint blockade resist tumor-mediated inhibition. J Clin Invest.

[145] Kloss CC, Lee J, Zhang A, Chen F, Melenhorst JJ, Lacey SF, Maus MV, Fraietta JA, Zhao Y, June CH (2018). Dominant-Negative TGF-β Receptor Enhances PSMA-Targeted Human CAR T Cell Proliferation And Augments Prostate Cancer Eradication. Mol Ther.

[146] Zhang Q, Helfand BT, Carneiro BA, Qin W, Yang XJ, Lee C, Zhang W, Giles FJ, Cristofanilli M, Kuzel TM (2018). Efficacy Against Human Prostate Cancer by Prostate-specific Membrane Antigen-specific, Transforming Growth Factor-β Insensitive Genetically Targeted CD8 T-cells Derived from Patients with Metastatic Castrate-resistant Disease. Eur Urol.

[147] Yamamoto TN, Lee P-H, Vodnala SK, Gurusamy D, Kishton RJ, Yu Z, Eidizadeh A, Eil R, Fioravanti J, Gattinoni L (2019). T cells genetically engineered to overcome death signaling enhance adoptive cancer immunotherapy. J Clin Invest.

[148] Kaur RP, Vasudeva K, Singla H, Benipal RPS, Khetarpal P, Munshi A (2018). Analysis of pro- and anti-inflammatory cytokine gene variants and serum cytokine levels as prognostic markers in breast cancer. J Cell Physiol.

[149] Freeman BE, Hammarlund E, Raué H-P, Slifka MK (2012). Regulation of innate CD8+ T-cell activation mediated by cytokines. Proc Natl Acad Sci U S A.

[150] Leen AM, Sukumaran S, Watanabe N, Mohammed S, Keirnan J, Yanagisawa R, Anurathapan U, Rendon D, Heslop HE, Rooney CM (2014). Reversal of tumor immune inhibition using a chimeric cytokine receptor. Mol Ther.

[151] Mohammed S, Sukumaran S, Bajgain P, Watanabe N, Heslop HE, Rooney CM, Brenner MK, Fisher WE, Leen AM, Vera JF (2017). Improving chimeric antigen receptor-modified T cell function by reversing the immunosuppressive tumor microenvironment of pancreatic cancer. Mol Ther.

[152] Bajgain P, Tawinwung S, D'Elia L, Sukumaran S, Watanabe N, Hoyos V, Lulla P, Brenner MK, Leen AM, Vera JF (2018). CAR T cell therapy for breast cancer: harnessing the tumor milieu to drive T cell activation. J Immunother Cancer.

[153] Wang Y, Jiang H, Luo H, Sun Y, Shi B, Sun R, Li Z (2019). An IL-4/21 Inverted Cytokine Receptor Improving CAR-T Cell Potency in Immunosuppressive Solid-Tumor Microenvironment. Front Immunol.

[154] Hartley J, Abken H (2019). Chimeric antigen receptors designed to overcome transforming growth factor-β-mediated repression in the adoptive T-cell therapy of solid tumors. Clin Transl Immunol.

[155] Liu X, Ranganathan R, Jiang S, Fang C, Sun J, Kim S, Newick K, Lo A, June CH, Zhao Y (2016). A Chimeric Switch-Receptor Targeting PD1 Augments the Efficacy of Second-Generation CAR T Cells in Advanced Solid Tumors. Cancer Res.

[156] Kobold S, Grassmann S, Chaloupka M, Lampert C, Wenk S, Kraus F, Rapp M, Düwell P, Zeng Y, Schmollinger JC (2015). Impact of a new fusion receptor on PD-1-mediated immunosuppression in adoptive T cell therapy. J Natl Cancer Inst.

[157] Rataj F, Kraus FBT, Chaloupka M, Grassmann S, Heise C, Cadilha BL, Duewell P, Endres S, Kobold S (2018). PD1-CD28 Fusion Protein Enables CD4+ T Cell Help for Adoptive T Cell Therapy in Models of Pancreatic Cancer and Non-hodgkin Lymphoma. Front Immunol.

[158] Park HB, Lee JE, Oh YM, Lee SJ, Eom H-S, Choi K (2017). CTLA4-CD28 chimera gene modification of T cells enhances the therapeutic efficacy of donor lymphocyte infusion for hematological malignancy. Exp Mol Med.

[159] Shin JH, Park HB, Oh YM, Lim DP, Lee JE, Seo HH, Lee SJ, Eom HS, Kim I-H, Lee SH (2012). Positive conversion of negative signaling of CTLA4 potentiates antitumor efficacy of adoptive T-cell therapy in murine tumor models. Blood..

